# Deletions within the 3′ Non-Translated Region of *Alfalfa mosaic virus* RNA4 Do Not Affect Replication but Significantly Reduce Long-Distance Movement of Chimeric *Tobacco mosaic virus*

**DOI:** 10.3390/v5071802

**Published:** 2013-07-17

**Authors:** Gourgopal Roy, Oleg Fedorkin, Masaaki Fujiki, Marina Skarjinskaia, Elisabeth Knapp, Shailaja Rabindran, Vidadi Yusibov

**Affiliations:** Fraunhofer USA Center for Molecular Biotechnology, 9 Innovation Way, Newark, DE 19711, USA; E-Mails: gourgopalroy@gmail.com (G.R.); ofedorkin@gmail.com (O.F.); fujikim@fdi.com (M.F.); mskarjinskaia@fraunhofer-cmb.org (M.S.); shailaja.rabindran@aphis.usda.gov (S.R.)

**Keywords:** *Alfalfa mosaic virus*, *Tobacco mosaic virus*, origin of assembly, subgenomic RNA, virus encapsidation

## Abstract

*Alfalfa mosaic virus* (AlMV) RNAs 1 and 2 with deletions in their 3′ non‑translated regions (NTRs) have been previously shown to be encapsidated into virions by coat protein (CP) expressed from RNA3, indicating that the 3′ NTRs of RNAs 1 and 2 are not required for virion assembly. Here, we constructed various mutants by deleting sequences within the 3′ NTR of AlMV subgenomic (sg) RNA4 (same as of RNA3) and examined the effect of these deletions on replication and translation of chimeric *Tobacco mosaic virus* (TMV) expressing AlMV sgRNA4 from the TMV CP sg promoter (Av/A4) in tobacco protoplasts and *Nicotiana benthamiana* plants. While the Av/A4 mutants were as competent as the wild-type Av/A4 in RNA replication in protoplasts, their encapsidation, long-distance movement and virus accumulation varied significantly in *N. benthamiana*. These data suggest that the 3′ NTR of AlMV sgRNA4 contains potential elements necessary for virus encapsidation.

## 1. Introduction

Encapsidation of the viral genome is an essential step in the life cycle of many plant viruses because efficient virus infection and spreading require production of virions. Packaging of the viral genome occurs following specific interactions between viral RNA and coat protein (CP). However, RNA elements involved in encapsidation and thus representing the origin of assembly (OAS) have been investigated for only a few viruses. The OAS of *Tobacco mosaic virus* (TMV) has been studied most extensively and shown to be located within the movement protein (MP)-coding sequence [1,2]. The OAS has been also identified in *Brome mosaic virus* [3,4], *Citrus tristeza virus* [5], *Turnip crinkle virus* [6] and *Peanut clump virus* [7]. 

Until now, identification of the OAS in the tripartite *Alfalfa mosaic virus* (AlMV), a member of the *Bromoviridae* family, has been elusive. Assembled AlMV forms four major bacilliform-shaped particles of 56 nm, 43 nm, 35 nm and 30 nm, depending on the size of the encapsidated RNA [8]. Each RNA molecule is encapsidated separately and all particles have the same diameter of 18 nm. RNAs 1 and 2 encode P1 and P2 proteins, respectively, which are required for replication of viral RNA [9,10]. RNA3 encodes MP (also known as P3), which is required for cell-to-cell movement [11], and CP [12]. CP is also translated from subgenomic RNA4 (sgRNA4) produced during infection. The 5′ and 3′ non‑translated regions (NTRs) of these four RNAs are highly structured and contain elements that are important for replication and translation. CP of AlMV has multiple functions including genome activation [13], regulation of plus- and minus-strand RNA synthesis [10,14], virus assembly [15], RNA stability [16,17], and long-distance movement [10,18]. Previous studies [19] have shown that RNAs 1 and 2 that had deletions in their 3′ NTRs were still encapsidated by CP expressed from RNA3. This indicates that the 3′ NTRs of RNAs 1 and 2 are not required for the virus assembly and that the elements required for encapsidation of these RNAs could be located within either 3′ NTR of RNA3 (or sgRNA4, since sgRNA4 is transcribed from RNA3) or within open reading frames (ORFs). A deletion analysis of the 3′ NTR sequence of AlMV RNA3 would be appropriate for locating specific RNA elements necessary for virus encapsidation. However, this would require engineering of viable AlMV mutants with altered levels of CP production, which is complicated due to diverse functionality of AlMV CP. 

**Figure 1:**
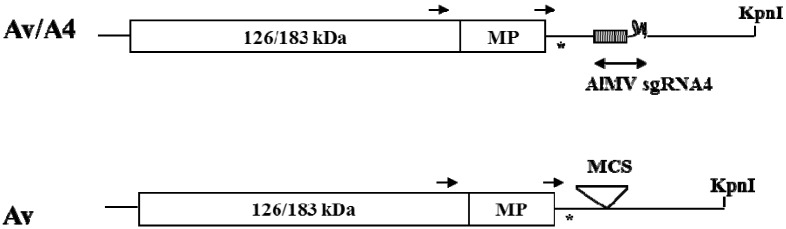
Schematic representation of the genomes of Av and Av/A4. Av is a *Tobacco mosaic virus* (TMV)-based expression vector that contains the TMV 5′ NTR, 126/183 kDa protein genes involved in replication, movement protein (MP) gene required for cell-to-cell movement and the 3′ NTR. The start codon for CP is mutated (indicated by an asterisk) and the coat protein (CP) open reading frame (ORF) is dissected by a multiple cloning site (MCS). Av/A4 contains the *Alfalfa mosaic virus* (AlMV) sgRNA4 sequence in the MCS of Av, so that this sequence is under the control of the TMV U1 CP sg promoter. Arrows indicate positions of MP and CP sg promoters.

Alternatively, viral chimeras containing heterologous CP can be used for identifying the RNA elements necessary for encapsidation. For this purpose, in the TMV-AlMV chimeric virus, Av/A4 [20] (Figure 1), the TMV CP ORF was replaced with the AlMV CP sgRNA4 sequence that included the 5′ NTR, CP ORF and 179-nucleotide (nt) long 3′ NTR sequence. Av/A4 is known to infect systemically *Nicotiana benthamiana*, *Nicotiana tabacum* and *Spinacia oleracea*, and the infection required formation of virions [20]. These results indicated that Av/A4 may have all the elements necessary for the chimeric TMV genome to be encapsidated by AlMV CP. 

In this study, we have generated mutants (p0, p1, p3 and p8) of Av/A4 containing various deletions within the 3′ NTR of AlMV sgRNA4 to examine their effects on chimeric virus replication, encapsidation, movement and accumulation and to identify the RNA elements required for virus encapsidation.

## 2. Results and Discussion

### 2.1. Effect of Deletions within the 3′ NTR of AlMV sgRNA4 on Chimeric Virus Replication and CP Accumulation

**Figure 2:**
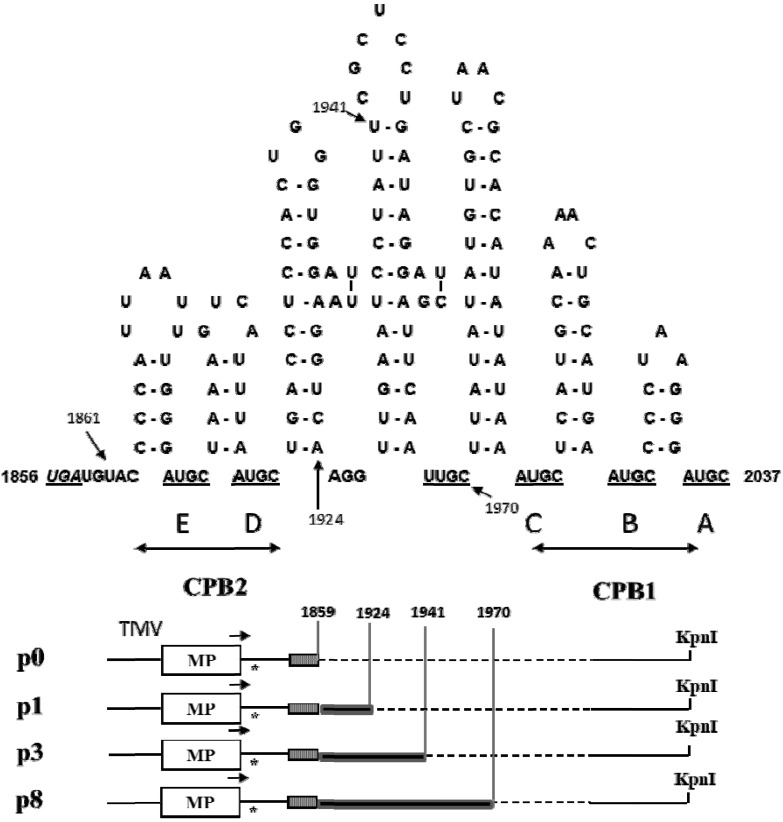
Construction of various deletions in CPB of Av/A4. Top: secondary structure of the 3′ non‑translated region (NTR) of AlMV RNA3 (or sgRNA4) in the CPB conformation. Hairpin loops in the 3′ NTR as shown by CPB1 and CPB2 are the CPB sites that are involved in replication and translation, respectively. Numbers indicate nt position in AlMV RNA3. Bottom: Av/A4 mutants p0, p1, p3 and p8 with respective deletion regions (shown by dotted lines within CPB regions). The lines upstream of MP represent sequences from TMV included in each of the Av/A4 mutants.

To investigate the effect of deletion of the AlMV sgRNA4 3′ NTR on replication of chimeric TMV (Av/A4), we generated Av/A4 mutants by deleting various length of sequences within CP binding site 2 (CPB2) and its downstream sequences, except from CPB1 which is needed for efficient RNA translation [21]. The mutants were p0 (no CPB2 domain is included), p1 (containing only CPB2), p3 (containing an extended CPB2 sequence) and p8 (containing a further extended CPB2 sequence) (Figure 2). 

**Figure 3:**
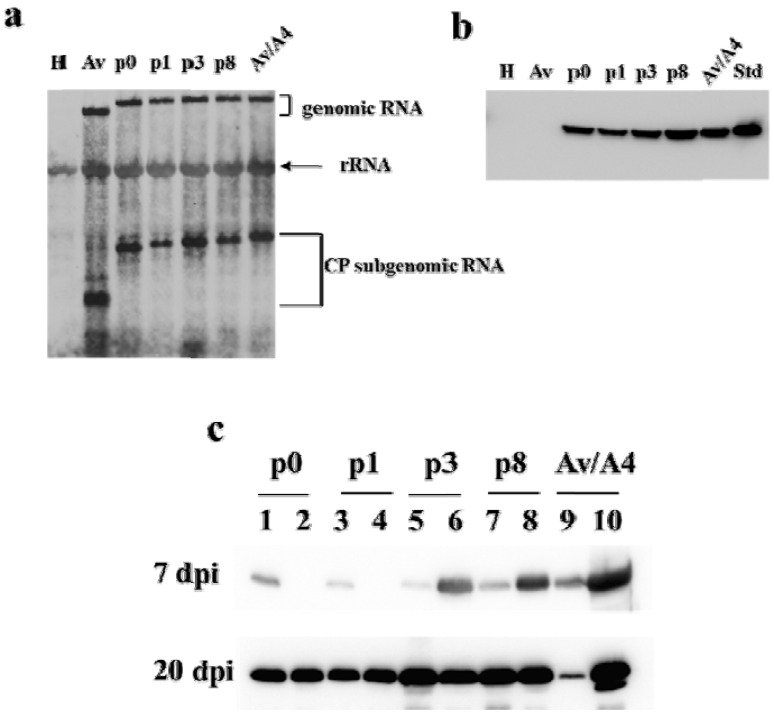
Examination of Av/A4 and deletion mutants in protoplasts and plants. (**a**) Northern blot analysis of total RNA isolated from tobacco protoplasts inoculated with *in vitro* transcripts of Av, p0, p1, p3, p8 or Av/A4 (as shown on blot) at 24 h post inoculation. A digoxigenin-labeled TMV 3′ NTR plus-strand–specific riboprobe was used for detection. Lane H indicates healthy protoplasts and rRNA is ribosomal RNA. (**b**) Western blot analysis of total protein from protoplasts inoculated with *in vitro* transcripts of each construct. Lanes are the same as in (**a**); Std indicates wild-type purified AlMV CP, 200 ng. (**c**) Western blot analysis of total protein from plants inoculated with *in vitro* transcripts of each construct. Tissue samples were obtained from inoculated (Lanes 1, 3, 5, 7 and 9) and upper non-inoculated leaves (Lanes 2, 4, 6, 8 and 10) at 7 and 20 days post inoculation (dpi). CP was detected by an anti-AlMV CP monoclonal antibody followed by a secondary antibody labeled with horseradish peroxidase.

Suspensions of tobacco protoplasts were inoculated with equivalent amounts of *in vitro* generated transcripts of each construct, and total RNAs were assessed by the Northern blot analysis at 24 h post inoculation. The results indicated that all the constructs produced genomic RNAs and CP sg mRNAs at comparable levels (Figure 3a). The construct Av also produced a similar level of CP sg RNA, but of a smaller size than the other constructs, which was expected since Av lacks the sequences of AlMV sgRNA4. Uninfected healthy protoplasts were used as a negative control and showed no detectable signal with the mobility of the genomic RNA or sgRNA in the Northern blot analysis, thus suggesting that the deletions within the AlMV sgRNA4 3′ NTR sequence did not affect chimeric Av/A4 virus replication or mRNA accumulation in protoplasts. 

The expression of CP from these constructs was also compared using the Western blot analysis. For that, total proteins were isolated from the transcript-inoculated protoplasts at 24 h post inoculation and probed with an anti-AlMV CP monoclonal antibody. The results demonstrated CP accumulation from all the mutants except Av. Among these, p3 and p8 showed a moderate to high level of CP accumulation compared to p0 and p1 (Figure 3b). Interestingly, both p0, which has a complete deletion of CPB2, and p1, which includes CPB2, showed similar levels of CP (Figure 3b). This suggests that inclusion or deletion of the CPB2 region (*i.e.*, p0 and p1 mutants) had no impact on CP accumulation, whereas inclusion of an extended (p3) and a further extended (p8) sequence downstream of CPB2 had a strong positive effect on CP accumulation (Figure 3b). 

Thus, the results indicate that, although AlMV mutants are as competent as Av/A4 in replication, they varied in CP accumulation in protoplasts. 

### 2.2. Symptom Expression, Encapsidation and Long-Distance Movement by AlMV sgRNA4 Mutants

Although the deletions within the AlMV sgRNA4 3′ NTR had no effect on chimeric virus replication in inoculated tobacco protoplasts, it was unknown whether the symptom expression in plants inoculated with these mutants would be similar. To address this question, we inoculated *N. benthamiana* plants with *in vitro* generated RNA transcripts of the mutants and monitored for symptom development. Plants infected with p0 or p1 did not develop any observable symptoms until 15–20 days post inoculation (dpi), whereas plants inoculated with p3 or p8, as well as with intact Av/A4, demonstrated visible curling and yellowing symptoms in upper non-inoculated leaves within 7 to 10 dpi. Moreover, these plants developed significant stunting. 

To examine whether symptomatic or asymptomatic plants contained AlMV CP, total proteins were extracted from upper systemic leaves of the infected plants at 7 and 20 dpi and analyzed by Western blotting (Figure 3c). The results demonstrated that at 7 dpi p3, p8 and Av/A4, but not p0 and p1, showed strong CP signals detected using an anti-AlMV CP monoclonal antibody (Figure 3c; compare lanes 6, 8 and 10 with lanes 2 and 4). Furthermore, CP signals were visible in leaves inoculated with any of these constructs (Figure 3c; lanes 1, 3, 5, 7 and 9), except from Av (data not shown). This suggests that at 7 dpi encapsidated virus particles of p3, p8 and Av/A4 moved efficiently into upper systemic leaves, while p0 and p1 mutants did not. However, at 20 dpi all the mutants, including p0 and p1, showed more or less equivalent CP signals in both inoculated as well as upper systemic leaves (Figure 3c), These results demonstrate that compared to p3, p8 and Av/A4, the mutants p0 and p1 were less efficiently encapsidated by CP, which affected their long-distance movement as evidenced by symptom expression and CP accumulation in upper systemic leaves of the inoculated plants at 7 dpi. 

Reduced encapsidation of p0 and p1 was further confirmed by passaging infectivity from the p0- and p1-infected plant sap to the local lesion host, *N. tabacum* cv. Xanthi NN plants, and counting the number of lesions on each inoculated leaf at 2 to 3 dpi. We have found that the mutants p3 and p8 passaged infectivity at a high efficiency (60 to 65%, similar to intact Av/A4), whereas p0 and p1 did it at a very low efficiency (less than 20%). This marked reduction in the efficiency of passaging with p0 and p1 suggests low efficiency in encapsidation for these chimeric constructs compared to p3, p8 and Av/A4.

Taken together, our findings indicate that deletions of the extended part of the CPB2 region (from nt position 1924 to 1970; Figure 2) of the 3′ NTR of sgRNA4, as in the p0 and p1 mutants, impaired the ability of viral RNA to be encapsidated efficiently and thus resulted in poor host infection and long-distance movement of the encapsidated virus particles. In contrast, the deletions in the p3 and p8 mutants did not have substantial effect.

### 2.3. Characterization of Purified Mutant Virus Particles

**Figure 4:**
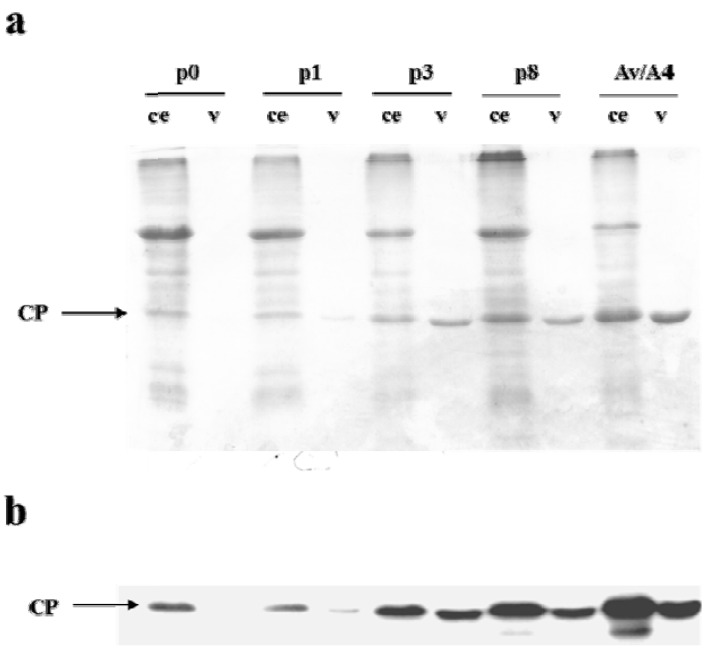
Analysis of virus preparations and the crude extracts by SDS-PAGE followed by (**a**) Coomassie staining and (**b**) Western blot analysis using an anti-AlMV CP antibody. *N. benthamiana* plants were inoculated with equivalent amounts of *in vitro* transcripts of p0, p1, p3, p8 or Av/A4. Virus was purified from upper non-inoculated leaves of the plants at 3 to 4 weeks post inoculation from equivalent amounts of tissue. Total protein present in the crude extracts from equivalent amounts of tissue infected with these constructs was also examined. CE: crude extract, v: purified virus preparation.

In order to characterize virus particles formed by the deletion mutants of Av/A4, we have purified them from upper systemic leaves 3 weeks post inoculation, following a previously described procedure for purification of AlMV virions [22]. The presence of the virus in the purified preparations was demonstrated by SDS-PAGE followed by Coomassie staining (Figure 4a) and the Western blot analysis (Figure 4b). We also assessed the virus yield from plants infected with the AlMV sgRNA4 3′ NTR deletion mutants. The comparative analysis of total protein in crude plant extracts and purified virus preparations demonstrated that the amount of purified particles from plants infected with p3, p8 or Av/A4 was quantitatively high compared with p0- and p1-infected plants (Figure 4a,b), whereas the yield of p3 and p8 was ~2-fold lower compared with Av/A4-infected plants. In contrast, the yield of p0- and p1-produced particles was negligible (~1% of the yield of Av/A4), although AlMV CP was detected in both inoculated and upper systemic leaves at 20 dpi (Figure 3c). 

**Figure 5:**
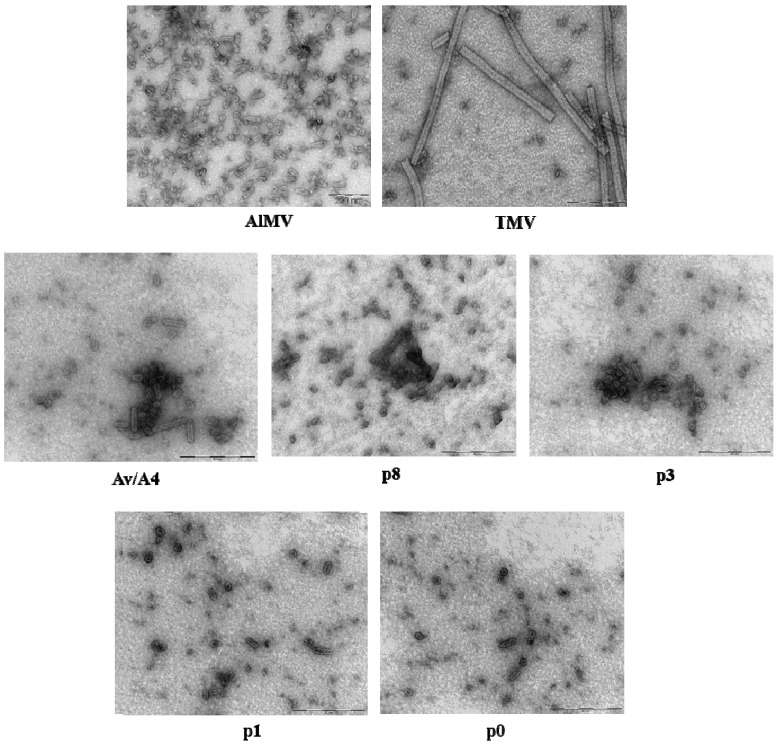
Transmission electron microscopy (TEM) analysis of virus particles purified from plants inoculated with *in vitro* transcripts of AlMV, TMV, Av/A4, p8, p3, p1 or p0.

We characterized the purified mutant virus preparations by transmission electron microscopy (TEM). The wild-type AlMV virus preparation typically contains bacilliform-shaped particles of four major sizes—56, 43, 35 or 30 nm long and 18 nm wide (Figure 5), whereas TMV assembles into rigid rods that are 300 nm long and 18 nm wide (Figure 5). Preparations of the Av/A4 hybrid virus contain only AlMV-like bacilliform-shaped particles, but no TMV-like rigid rods (Figure 5). These preparations are very heterogeneous and contain particles of at least six different sizes—95, 70, 62, 44–48, 30–35 or 16–24 nm long, and ~18 nm wide (Figure 5, Table 1). Approximately 10% of the particles were longer than 62 nm, and ~90% were in the 16–48 nm length range. The preparation of the p8 mutant contained virus particles similar to the virions of intact Av/A4, whereas in the preparation of the mutant p3 we could detect only 62 nm or shorter particles and none longer than 70 and 95 nm (Figure 5, Table 1). Furthermore, in the preparations of the p0 and p1 mutants we observed even shorter particles, 16–48 nm long. 

Taken together, our purification results and TEM observations indicated that the longer the sequence deleted from the 3′ NTR of AlMV sgRNA4, the lower the yield of the mutant virus particles and the shorter their length.

**Table 1:** Distribution of virus particles in purified virus preparations of Av/A4, p8, p3, p1 and p0.

Construct	Particle size (nm) ^a^					
	95 nm	70	62	44–48	30–35	16–24
Av/A4	+	+	+	+	+	+
P8	+	+	−	+	+	+
P3	−	−	+	+	+	+
P1	−	−	−	+	+	+
P0	−	−	−	+	+	+

^a^ Due to the presence of particles of different sizes in the virus preparations, similar-size particles were grouped together.

The results of the presented study, using tobacco protoplasts and *N. benthamiana* plants inoculated with the deletion mutants of Av/A4 chimeric TMV virus, have demonstrated that the 3′ NTR of AlMV sgRNA4 is not essential for replication of Av/A4 but is required for efficient RNA encapsidation, long-distance movement and virus accumulation. Our long-term goal is to identify the AlMV OAS which appears to be within the 3′ NTR of sgRNA4 based on the current findings. However, additional research with more compelling evidence is needed to demonstrate this. In this study, we used the Av/A4 chimeric virus as a model, because it allows for studying functions of CP and 3′ NTR sequences of AlMV sgRNA4 independently. This has bypassed the need for creating viable CP mutants using AlMV RNAs 1, 2 and 3. Since the OAS of many viruses typically consists of stem-loop structures [1,5,23] and the 3′ NTR of AlMV sgRNA4 (and RNA3) is highly structured, we examined the role of the 3′ NTR in encapsidation of Av/A4. For this purpose, we have generated mutants of Av/A4 that differed in the length of the sequences deleted from the 3′ NTR and explored their ability to replicate in protoplasts, encapsidate RNA and infect plants. 

Because the sequences required for Av/A4 encapsidation were found to localize within the AlMV 3′ NTR, we hypothesized that Av/A4 would form at least three types of major-size virus particles. These particles were expected to encapsidate genomic RNA (~7,350 nts), MP sgRNA (~2,500 nts) or CP sgRNA (~1,650 nts). The TEM analysis of purified Av/A4 demonstrated the presence of bacilliform-shaped particles with rounded ends typical for AlMV, rather than the rigid rods typical for TMV. Moreover, the Av/A4 virus preparation was more heterogeneous than expected, showing at least six types of particles of different sizes (Table 1). When similar-size particles were grouped together (shorter particles, 16–48 nm; longer particles, 62–95 nm), the shorter particles predominated (~90%), suggesting that encapsidation of the longer particles in the Av/A4 virus preparation was relatively inefficient (10%), which could be due to the large size of the Av/A4 genome (~7,350 nts, twice as the size of AlMV RNA1 [3,644 nts; the particle size, 56 nm]). 

Deletion of certain elements of the 3′ NTR has been also found to have an effect on the efficiency of encapsidation and size of Av/A4 mutant s and their ability to infect plants. Although the shorter particles (16–48 nm) were observed even in the purified preparations of the mutants p0 and p1, these preparations may have infected plants very inefficiently, indicating poor encapsidation of these mutants. Although we did not examine the encapsidated RNA, it is also possible that p0 or p1 could form empty particles, and this may be the reason why we observed CP expression on a Western blot. Studies with a TMV-BMV hybrid [3] in which *Brome mosaic virus* CP replaced native CP revealed that in the hybrid virus only three sgRNAs, but not genomic RNA, were encapsidated. CP or sequences and/or structural features within the chimeric viral genome were thought to play a role in promoting virion assembly [3].

## 3. Experimental Section

### 3.1. DNA Constructs

Cloning procedures were performed according to [24]. *Escherichia coli* DH5α cells (Life Technologies, Gaithersburg, MD, USA) were used for transformation. Av and Av/A4 constructs (Figure 1) have been described previously [20]. To construct p0 that does not have the AlMV sgRNA4 3′ NTR, the EcoRI-Rsa I fragment of pSP65A4 [16] containing the 5′ NTR of sgRNA4 and the CP ORF was blunt-ended and then ligated into the XhoI site of Av [20]. Constructs p1, p3 and p8 containing the 5′ 66, 83, and 112 nts of the 3′ NTR, respectively (in the AlMV RNA3, positions 1859 to 1924, 1859 to 1941 and 1859 to 1970, respectively), were created using PCR and appropriate primers, and the PCR fragments were digested with NcoI and SalI and cloned into Av/A4 digested with NcoI and XhoI.

### 3.2. *In Vitro* Transcription and Protoplast Inoculation

Plasmids were linearized with KpnI before performing *in vitro* transcription reactions. *In vitro* transcripts of each construct were synthesized using the Amplicap T7 kit (Epicentre Biotechnologies, Madison, WI, USA). Protoplasts were prepared from a *N. tabacum* cv. Xanthi suspension cell line and inoculated using procedures described previously [25].

### 3.3. Plant Inoculation and Virus Purification

*N. benthamiana* plants were inoculated with *in vitro* transcripts of various constructs as described previously [20]. Virus was purified from the infected tissue two to three weeks post inoculation using procedures described previously [26]. Briefly, the leaf tissue was ground and the sap was separated from the cell debris by centrifugation. Virus particles were selectively precipitated by 5% polyethylene glycol (MW 20,000). TMV was purified from infected plants by procedures described previously [27]. 

### 3.4. Northern Blot Hybridization

Northern blot hybridization of total nucleic acid extracted from protoplasts or virion RNA was performed as described previously [25], using a plus-strand–specific digoxigenin-labeled riboprobe specific to the 3′ NTR of TMV. 

### 3.5. Protein Analysis

Total protein from protoplasts or plants was analyzed by SDS-PAGE followed by Western blotting using a monoclonal antibody against AlMV CP (Agdia, Elkhart, IN, USA). The blots were then incubated with a secondary antibody labeled with horseradish peroxidase and incubated with the enhanced chemiluminescence reagent (ECL; Pierce Biotechnology, Rockford, IL, USA). Chemiluminescence was detected and immunoblot images were obtained using the Gene Gnome Imaging System supported by the Gene Tools software package (both from Syngene, Frederick, MD, USA). For the purified virus analysis, the gels were stained with Coomassie Brilliant blue and then scanned for documentation.

### 3.6. Examination of Virion Stability in Plant Sap

*N. benthamiana* plants were mechanically inoculated with *in vitro* transcripts of each construct. At 10 dpi, tissue from inoculated leaves was homogenized in 50 mM phosphate buffer (pH 6.5) and incubated at room temperature. Aliquots were taken at time 0 and 20 min after grinding. The inoculum was mechanically applied onto two half-leaves of *N. tabacum* cv. Xanti NN (a local lesion host). At 2 to 3 dpi the number of lesions was counted. The experiment was performed in triplicate and the averages were calculated.

### 3.7. Transmission Electron Microscopy

The TEM analysis of the purified virus preparation was performed using carbon-coated EM grids (Electron Microscope Science, Hatfield, PA, USA). Briefly, one drop of the virus preparation was placed on the grid for 1 min and then blotted with filter paper, leaving a small amount of liquid on the grid. A drop of uranyl acetate or 5% ammonium molybdate was placed on the grid, and after 30–45 s the grid was blotted with filter paper and allowed to dry. Samples were studied using the Zeiss CEM 902 TEM. Morphology characterization and size measurements of virus particles were performed using the ‘Analysis’ software from Soft Imaging Systems, Muenster, Germany. 

## 4. Conclusions

The results of our experiments with the Av/A4 deletion mutants suggest that the sequences within the 3′ NTR of AlMV sgRNA4 (and RNA3) are required for efficient encapsidation of the TMV/AlMV hybrid, Av/A4. Therefore, one may hypothesize that the sequences within 3′ NTR of AlMV sgRNA4 (and RNA3) play an important role in encapsidation of wild-type AlMV and also function *in trans* for encapsidation of AlMV RNAs 1 and 2. The latter is supported by the results of earlier studies which indicated that the 3′ termini of RNAs 1 and 2 are not required for the wild-type AlMV assembly [19]. The authors observed that mutants of RNAs 1 and 2 that had deletions within their respective 3′ NTRs could be still encapsidated by CP translated from RNA3 and suggested that the OAS of AlMV was therefore located either within the 3′ NTR of RNA3 or within the ORFs [19]. Other investigators demonstrated that the *cis*-acting 3′ tail-like structure (TLS) of BMV RNA3 was obligatory for encapsidation of BMV RNA3 but not RNAs 1 and 2. Therefore, when this 3′ TLS from BMV RNA3 was provided *in trans* to RNAs 1 and 2 that lacked their 3′ TLSs, these RNAs were encapsidated [28]. In addition to the TLSs in the 3′ NTRs of BMV RNAs, some elements essential for efficient packaging of BMV RNA3 have been identified within the BMV MP ORF [3,4]. These reports, along with the results of our study, warrant additional investigations to elucidate the elements necessary for encapsidation of AlMV. 
